# Comparison of Saffron and Fluvoxamine in the Treatment of Mild to Moderate Obsessive-Compulsive Disorder: A Double Blind Randomized Clinical Trial

**Published:** 2017-07

**Authors:** Sophia Esalatmanesh, Mojtaba Biuseh, Ahmad Ali Noorbala, Seyed-Ali Mostafavi, Farzin Rezaei, Bita Mesgarpour, Payam Mohammadinejad, Shahin Akhondzadeh

**Affiliations:** 1 Psychiatry and Psychology Research Center, Roozbeh Hospital, Tehran University of Medical Sciences, Tehran, Iran.; 2 Psychosomatic Research Center, Imam Khomeini Hospital, Tehran University of Medical Sciences, Tehran, Iran.; 3 Qods Hospital, Kurdistan University of Medical Sciences, Sanandaj, Iran.; 4 National Institute for Medical Research Development (NIMAD), Tehran, Iran.

**Keywords:** *Crocus Sativus*, *OCD*, *Saffron*, *Serotonin*, *Trial*

## Abstract

**Objective:** There are different pathophysiological mechanisms for obsessive- compulsive disorder (OCD) as suggested by the serotonergic, dopaminergic, and glutamatergic hypotheses. The present study aimed at comparing the efficacy and safety of saffron (stigma of Crocus sativus) and fluvoxamine in the treatment of mild to moderate obsessive- compulsive disorder.

**Method:** In this study, 50 males and females, aged 18 to 60 years, with mild to moderate OCD, participated. The patients were randomly assigned to receive either saffron (30 mg/day, 15 mg twice a day) or fluvoxamine (100 mg/day) for 10 weeks. Using the Yale-Brown Obsessive Compulsive Scale (Y-BOCS) and the Adverse Event Checklist, we assessed the patients at baseline, and at the second, fourth, sixth, eighth, and tenth week. Finally, the data were analyzed using general linear repeated measures.

**Results:** In this study, 46 patients completed the trial. General linear repeated measures demonstrated no significant effect for time-treatment interaction on the Y-BOCS total scores [F (2.42, 106.87) = 0.70, P = 0.52], obsession Y-BOCS subscale scores [F (2.47, 108.87) = 0.77, p = 0.49], and compulsion Y-BOCS subscale scores [F (2.18, 96.06) = 0.25, P = 0.79]. Frequency of adverse events was not significantly different between the 2 groups.

**Conclusion:** Our findings suggest that saffron is as effective as fluvoxamine in the treatment of patients with mild to moderate OCD.

Obsessive-compulsive disorder (OCD) is a common mental disorder with an estimated lifetime prevalence of 1% to 3% in the general population (1, 2). Emotional and social capacities and quality of life are severely compromised in OCD patients (3). Different pathophysiological mechanisms have been suggested for OCD via the serotonergic, dopaminergic, and glutamatergic hypotheses (4-7). Several studies have assessed the efficacy of SSRIs for OCD, and the results showed that they are more effective than placebo (8). 

Exposure and response prevention (ERP) and serotonin reuptake inhibitors (SRIs) are among the first line interventions in the treatment of OCD patients. Even with appropriate treatment, symptoms can wax and wane (9). High doses of selective serotonin reuptake inhibitors (SSRIs) can cause prolonged QT intervals and arrhythmias (10).

Several studies have shown that SSRIs are frequently associated with sexual dysfunction (11, 12). Herbal medications have shown less side effects and drug interactions compared to chemical drugs (13).

*Crocus sativus*, commonly known as saffron, belongs to the Iridaceae family. Saffron contains several compounds such as safranal (responsible for saffron odor and aroma), picrocrocin (responsible for bitter taste of saffron), and crocin (the main saffron antioxidant used as dye material). In folk medicine, saffron is used as an antispasmodic, eupeptic, gingival sedative, anti-catarrhal, nerve-sedative, carminative, diaphoretic, expectorant, stimulant, stomachic, and aphrodisiac (14). Saffron can affect the chemical neurotransmitters in the brain such as dopamine and glutamate (15). Previously, we have demonstrated the beneficial antidepressant and antidementia effects of saffron (16-20). A recent animal study has reported that active constituents of saffron, crocins, may play a role in compulsive behavior, supporting a functional interaction between crocins and the serotonergic system (21). To the best of our knowledge, no clinical study was done on the efficacy of saffron in the treatment of OCD. The present study was conducted to assess the efficacy and tolerability of saffron in the treatment of mild to moderate OCD patients compared to fluvoxamine.

## Materials and Methods

***Trial Setting and Design***

This was a single center, 10- week, randomized, double- blind, parallel group trial conducted in the outpatient clinic of Roozbeh psychiatric hospital (affiliated to Tehran University of Medical Sciences, Tehran, Iran) from December 2014 to May 2016. The trial was registered at the Iranian registry of clinical trials (www.irct.ir; registration number: IRCT201407151556N62) prior to the study. The trial protocol was approved by the institutional review board (IRB) of Tehran University of Medical Sciences (Grant No: 25406) and was conducted in accordance with the Declaration of Helsinki and subsequent revisions. All patients signed an informed consent prior to study entry. Patients were informed that they were free to withdraw from the trial at any time without any adverse effect on their therapy or the relationship with their health care provider.

***Participants***

Males and females aged 18 to 60 years, with a diagnosis of OCD according to the Diagnostic and Statistical Manual of Mental Disorders, Fourth Edition, Test Revision (DSM-V-TR) and a Yale-Brown Obsession Compulsion Scale (Y-BOCS) scores of 12 to 21 (mild to moderate OCD) were eligible to participate in the trial (22, 23). This was the first clinical study to assess the efficacy of saffron in the treatment of OCD patients; and mild to moderate OCD (scores 12 to 21) was selected according to the ethical issues. Exclusion criteria were as follow: any other mental disorder on the DSM- IV axis I, alcohol or substance (other than nicotine or caffeine) dependence, any serious medical illness including cardiac, hepatic, and renal diseases, neurologic diseases, pregnancy, and breast feeding. Participants did not receive any other psychiatric medications 6 weeks prior to the study. ***Interventions***

Patients underwent a standard clinical assessment that included a psychiatric evaluation, a structured diagnostic interview, and medical history. Eligible participants were randomized to receive either a capsule of saffron (SaffroMood®, Green Plant Life and containing 15 mg of saffron extract) twice daily, or a fluvoxamine capsule (Sobhan) 100 mg/day for 10 weeks. The stigma extract was standardized based on crocin by means of spectrophotometry. Crocin value is expressed as direct reading of the absorbance at about 440 nm. Each capsule had 1.65–1.75 mg crocin. Participants were not allowed to use any psychotropic drug or any behavioral intervention therapy during the course of the trial. 

***Outcome***

Y-BOCS was used to assess patients at baseline and at weeks 2, 4, 6, 8, and 10. Y-BOCS is a validated 10-item rating scale, which has been widely applied in psychiatric studies to measure the severity of OCD symptoms, and has also been used to assess the severity of obsessive and compulsive symptoms in several clinical trials in Iran (24-28). Furthermore, Rajezi Esfahani et al. have assessed the reliability and validity of the Persian version of Y-BOCS; and they found that its internal consistency, split-half reliability, and test-retest reliability coefficients varied from 0.89 to 0.99. Moreover, concurrent reliability were evaluated and approved by correlating the Y-BOCS with the SCL-90-R-OCS and SCID-I (29). The primary outcome measure was the difference in Y-BOCS total score change from baseline to the end of the trial between the 2 groups using the general linear repeated measure model. Secondary outcome measures were comparing changes in Y-BOCS total, obsession and compulsion subscale scores from baseline to week 10 between the 2 groups, partial response rates (≥ 25% reduction in the Y-BOCS score), and complete response rates (≥ 35% reduction in the Y-BOCS score)(30). Adverse events were recorded systematically at each visit using a checklist (31, 32). All participants were asked about any adverse event, which was not mentioned in the checklist. A thorough physical examination was performed at the screening session and at each postbaseline visit. Furthermore, a complete blood count (CBC) was taken, and serum aminotransferases and renal function test values were measured at baseline and at each postbaseline visit.

***Sample Size***

A sample size of 46 (each group 23) was calculated assuming a difference of 3 on Y-BOCS, a standard deviation (SD) of 3, a power of 80%, and a two- sided significance level of 5%. Considering a 10% attrition rate, a final sample size of 50 was achieved. 

 Randomization, Allocation Concealment, and Blinding

Generation of randomization codes was conducted by a computerized random number generator (blocks of 4, allocation ratio 1:1) by an independent party, who was not involved elsewhere in the trial. Allocation was concealed using successively numbered, opaque, and sealed envelopes. Saffron and fluvoxamine were identical in their shape, size, texture, color, and odor. The patients, research investigator, and the rater were all blind to the treatment allocation.

***Statistical Analysis***

Continuous variables were reported as mean ± SD, and categorical variables were reported as frequency (percentage). Independent t test was used to compare baseline continuous variables. The mean difference between the saffron and fluvoxamine group was reported as mean difference [MD, 95% confidence interval (CI)]. A general linear model repeated measure was used to evaluate time-treatment interaction considering the treatment groups (saffron vs. fluvoxamine) as the between subject factor, and the study measurement as the within subject factor (time).Whenever Mauchly's test of sphericity was significant; Greenhouse-Geisser adjustment was used for degrees of freedom. An independent t test and Cohen's d effect size were used to compare score change from baseline to each time interval between the 2 groups. Categorical variables were compared using Chi-square or Fisher's exact test where appropriate. All analyses were performed two-sided, and a p-value of less than 0.05 was considered statistically significant. Statistical Package of Social Science Software (SPSS Version 22; IBM Company, USA) was used for statistical analysis. 

## Results

***Patients***

**Figure1:**
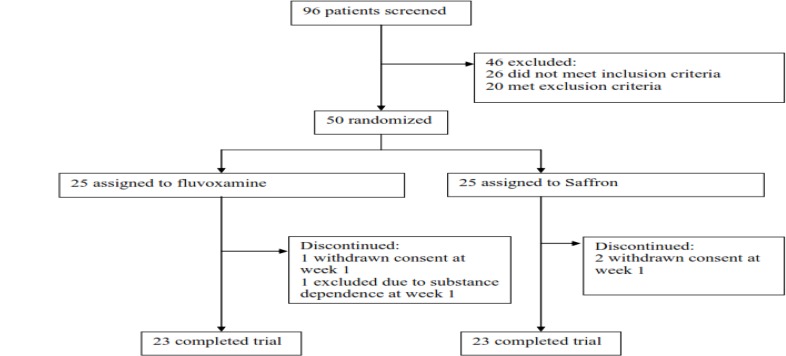
Flow diagram of the study

Among 96 patients, who were screened for the eligibility criteria, 50 patients were entered into the trial and randomized to receive either saffron (n = 25) or fluvoxamine (n = 25). Four patients discontinued the trial (2 patients in each group before Week 2), and a total number of 46 patients (23 in each group) completed the trial (Figure1). Baseline characteristics of the participants are summarized in Table 1.

***Y-BOCS Total Score***

**Figure2:**
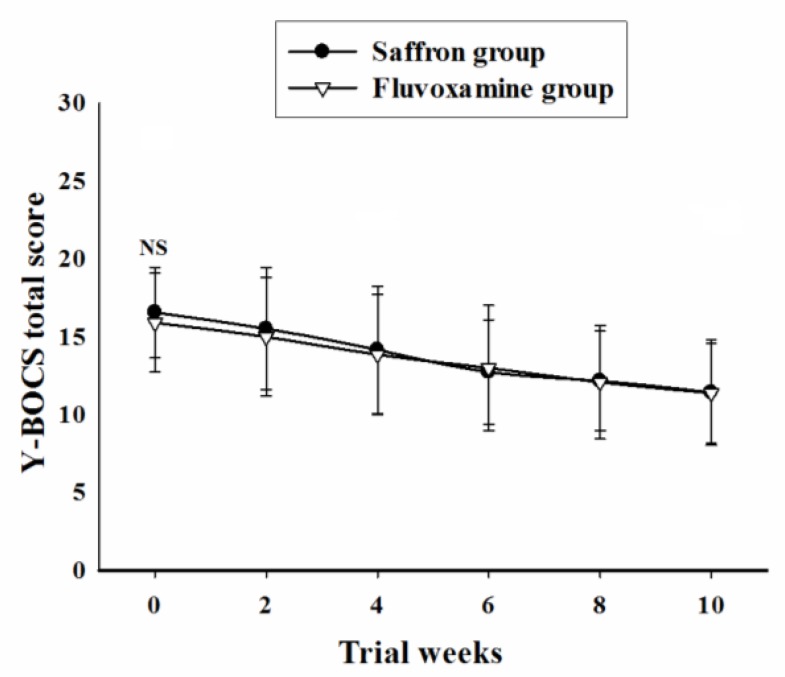
Repeated measure analysis of variance for the effect of two treatments on the Yale-Brown Obsessive Compulsive Scale (Y-BOCS) total scores. P-values show the result of the independent t-test for comparison of scores between the two groups at each time interval (mean ± SD; NS, not significant).

Baseline Y-BOCS total score did not differ significantly between the 2 groups [MD (95% CI) = 0.65 (-1.15 to 2.46), t (44) = 0.72, P = 0.47]. General linear model repeated measures demonstrated no significant effect for time-treatment interaction on Y-BOCS total score during the trial course [F (2.42, 106.87) = 0.70, P = 0.52] (Figure 2). Partial and complete response rates did not differ significantly between the 2 groups at the end of the trial (Table 2).

**Table1:** Baseline Characteristics According to the Treatment Group (Saffron and Fluvoxamine)

	**Saffron ** **group(n=23)**	**Fluvoxamine ** **group(n=23)**	**P-value**
Age, years, mean ± SD	34.00 ± 7.14	31.22 ± 7.27	0.19
Sex, female, n (%)	15 (65.2%)	13 (56.5%)	0.54
duration of the disease (years), mean ± SD	10.26 ± 5.56	9.39 ± 5.18	0.58
**Education**			0.33
Illiterate	5 (21.7%)	1(4.3%)	Non significant
primary	5 (21.7%)	7(30.4%)	
Middle	5 (21.7%)	6(26.1%)	
High school	4(17.4%)	2(8.7%)	
Higher	4(17.4%)	7(30.4%)	
Smoking, n, %	8(34.8%)	7(30.4%)	0.75
**Marital status**			0.17
Married	9(39.1%)	13(56.5%)	Non significant
Single	13(56.5%)	7(30.4%)	
Separated	1(4.3%)	3(13%)	
Y-BOCS total score, mean ± SD	16.57 ± 2.90	15.91 ± 3.17	0.47
Y-BOCS obsession subscale, mean ± SD	9.17 ± 2.85	9.26 ± 3.27	0.92
Y-BOCS compulsion subscale, mean ± SD	7.39 ± 2.10	6.65 ± 2.70	0.30

**Table2:** Comparison of Outcome Indexes between the Two Groups (Saffron and Fluvoxamine)

	**Saffron group**	**Fluvoxamine group**	**P-value**	**Odd’s ** **ratio(95%CI)**
Number (%) of partial response at weak 2	3(13%)	1(4.3%)	0.60	3.30(0.31-34.35)
Number (%) of partial response at weak 4	3(13%)	4(17.4%)	1.00	0.71(0.14-3.61)
Number (%) of partial response at weak 6	14(60.9%)	6(26.1%)	0.01	4.40(1.26-15.41)
Number (%) of partial response at weak 8	17(73.9%)	14(60.9%)	0.34	1.82(0.52-6.37)
Number (%) of partial response at weak 10	16(69.56%)	15(65.2%)	1.00	1.92(0.51-7.12)
Number (%) of Complete response at weak 2	0(0%)	0(0%)	-	-
Number (%) of Complete response at weak 4	3(13%)	2(8.7%)	1.00	1.57(0.23-10.43)
Number (%) of Complete response at weak 6	4(17.4%)	4(17.4%)	1.00	1.00(0.21-4.59)
Number (%) of Complete response at weak 8	5(21.7%)	4(17.4%)	1.00	1.31(0.30-5.70)
Number (%) of Complete response at weak 10	8(34.7%)	7(30.4%)	1.00	1.46(0.43-4.98)

**Table3:** Comparison of Changes in the Yale-Brown Obsessive Compulsive Scale Total and Subscales Scores from Baseline between the Two Groups (Saffron and Fluvoxamine) Using Independent T-Test

**Y-BOCS score**	**Saffron ** **group(n=23)**	**Fluvoxamine ** **group(n=23)**	**Mean difference ** **saffron- fluvoxamine ** **(95%CI)**	**Cohen’s d**	**P-value**
total (weak 2)	1.04 ± 1.94	0.91 ± 1.12	0.13 ( -0.81 to 1.07)	0.08	0.78
total (weak 4)	2.39 ± 2.48	2.04 ± 1.63	0.34 (-0.90 to 1.59)	0.16	0.57
total (weak 6)	3.82 ± 2.47	2.91 ± 2.04	0.91 (-0.43 to 2.26)	0.40	0.18
total (weak 8)	4.39 ± 2.75	3.82 ± 2.30	0.56 (-0.94 to 2.07)	0.22	0.45
total (weak 10)	5.13 ± 2.98	4.52 ± 2.52	0.60 (-1.03 to 2.25)	0.22	0.45
0bsession(weak2)	0.69 ± 1.42	0.73 ± 0.81	-0.04 (-0.73 to 0.64)	0.03	0.90
0bsession(weak4)	1.65 ± 1.77	1.60 ± 1.03	0.04 (-0.81 to 0.90)	0.03	0.92
0bsession(weak6)	2.82 ± 1.66	2.26 ± 1.35	0.56 (-0.33 to 1.46)	0.37	0.21
0bsession (weak 8)	3.26 ± 2.00	2.82 ± 1.46	0.43 (-0.60 to 1.47)	0.25	0.40
0bsession (weak 10)	3.86 ± 2.34	3.43 ± 1.75	0.43 (-0.79 to 1.66)	0.20	0.48
compulsion (weak 2)	0.34 ± 1.15	0.17 ± 0.57	0.17 (-0.37 to 0.72)	0.18	0.52
compulsion (weak 4)	0.69 ± 1.49	0.43 ± 0.84	0.26 (-0.45 to 0.98 )	0.21	0.46
compulsion (weak 6)	1.62 ±1.0	0.65 ± 1.19	0.34 (-0.49 to 1.19 )	0.24	0.41
compulsion (weak 8)	1.13 ± 1.65	1.00 ± 1.50	0.13 (-0.81 to 1.07)	0.08	0.78
compulsion (weak 10)	1.26 ± 1.62	1.08 ± 1.62	0.17 (-0.79 to 1.13)	0.11	0.71

**Table4:** The Frequency of Adverse Events in Patients with OCD receiving Saffron or Fluvoxamine

**Adverse event, n, %**	**Saffron** **group(n=23)**	**Fluvoxamine ** **group(n=23)**	**P-value**
headache, n, %	2(8.7%)	3(13%)	1.00
Dry mouth, n, %	3(13%)	3(13%)	1.00
nausea, n, %	3(13%)	3(13%)	1.00
Daytime drowsiness, n, %	2(8.7%)	2(8.7%)	1.00
constipation, n, %	2(8.7%)	3(13%)	1.00
sweating, n, %	2(8.7%)	4(17.4%)	0.66
vomiting, n, %	2(8.7%)	3(13%)	1.00

***Y-BOCS Obsession Subscale Score***

**Figure3:**
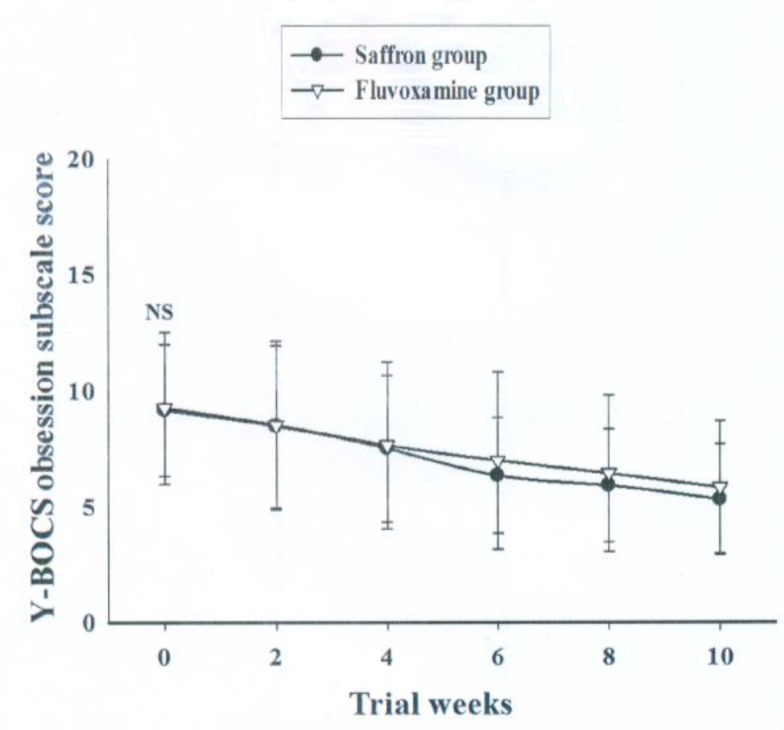
Repeated measure analysis of variance for the effect of two treatments on the Yale-Brown Obsessive Compulsive Scale (Y-BOCS) obsession subscale scores. P-values show the result of the independent t-test for comparison of scores between the two groups at each time interval (mean ± SD; NS, not significant).

Baseline Y-BOCS obsession subscale score did not differ significantly between the 2 groups [MD (95% CI) = -0.08 (-1.91 to 1.73), t (44) = -0.09, p = 0.92]. General linear model repeated measure demonstrated no significant effect for time-treatment interaction on Y-BOCS total score [F (2.47, 108.87) = 0.77, p = 0.49] (Figure 3). The reduction in Y-BOCS obsession subscale score did not differ significantly between the 2 groups at weeks 2, 4, 6, 8, and 10 ( Table 3(

***Y-BOCS Compulsion Subscale Score***

**Figure4:**
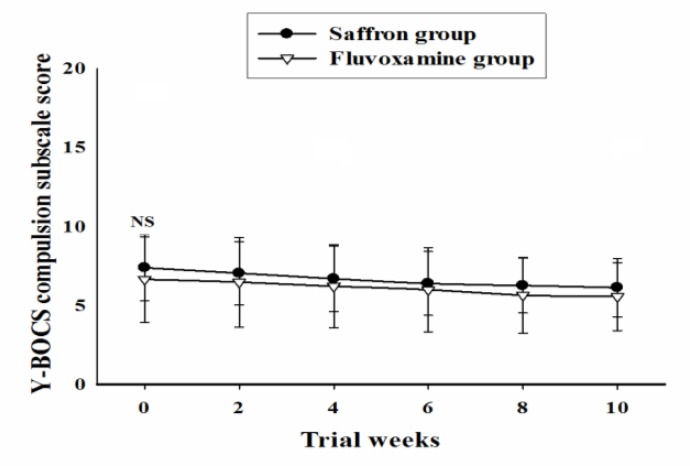
Repeated measure analysis of variance for the effect of two treatments on the Yale-Brown Obsessive Compulsive Scale (Y-BOCS) compulsion subscale scores. P-values show the result of the independent t-test for comparison of scores between the two groups at each time interval (mean ± SD; NS, not significant).

Baseline Y-BOCS compulsion subscale scores did not differ significantly between the 2 groups [MD (95% CI) = 0.73 (-0.70 to 2.18), t (44) = 1.03, p = 0.30]. General linear model repeated measure demonstrated no significant effect for time-treatment interaction on Y-BOCS total score [F (2.18, 96.06) = 0.25, P = 0.79] (Figure 4). The reduction in Y-BOCS compulsion subscale score did not differ significantly between the 2 groups at weeks 2, 4, 6, 8, and 10 ( Table 3).

***Adverse Events***

The frequency of adverse events did not differ significantly between treatment groups (Table 4). No serious adverse event and no death occurred.

## Discussion

The present study, to the best our knowledge, was the first double- blind study on the efficacy of saffron in the treatment of mild to moderate OCD patients. It provides evidence for satisfactory outcomes with saffron in the treatment of mild to moderate OCD (15 mg, 2 capsules per day). In this double-blind randomized clinical study of *Crocus sativus* stigma vs. fluvoxamine in the treatment of mild to moderate OCD, *Crocus sativus* was demonstrated to be as safe and effective as fluvoxamine for up to 10 weeks after the treatment initiation. Different pathophysiological mechanisms have been considered for OCD including the serotonergic, dopaminergic, and glutamatergic mechanisms (4-6). There is a growing body of evidence suggesting the important role of serotonin (5HT) reuptake inhibitor antidepressants in the treatment of OCD (5). Treatment with clomipramine was reported to reduce the concentration of 5-hydroxyindoleacetic acid (5-HIAA), the major central metabolite of 5HT, in the cerebrospinal fluid (32). In a rat model, a study on the role of striatum in compulsive behavior was conducted and revealed that a primary pathology of the orbitofrontal cortex may lead to dysregulation of the striatal serotonergic system in a subpopulation of OCD patients (33). The serotonergic (5-HT) system plays a major role in the action of antidepressants. Wang et al. reported that *Crocus sativus* corms extract is an effective antidepressant medicinal plant material (34). Previously, we indicated the antidepressant effect of saffron (16, 18 and 35). The antidepressant effects of saffron are potentially due to its serotonergic, antioxidant, anti-inflammatory, neuroendocrine and neuroprotective effects (36). Talaei et al. suggested that the antidepressant effect of saffron extract could be attributed to crocin as the main antioxidant constituent in saffron. In this 4- week trial, addition of crocin tablets (30 mg/day, 15 mg BID) amplified the effects of SSRIs in the treatment of patients with mild to moderate depression (37). Agha-Hosseini et al. indicated the efficacy of saffron in improving depression scores compared to the placebo control group in females with regular menstrual cycles, who experienced premenstrual syndrome (17). Amin et al. reported that crocin and crocetin, 2 major active components of *Crocus sativus*, produced antidepressant-like effect in forced swim test (FST) in mice (38). 

Saffron extract may inhibit serotonin reuptake in synapses. A study on an animal model of obsession compulsion disorder, suggested that crocin may alleviate the mCPP- induced excessive OCD-like behavior (nonselective serotonin (5-HT) receptor agonist) by an antagonist action at the 5-HT2C receptor site and may also support functional interactions between crocins and the serotonergic systems (21). Mohamadpour et al. evaluated the safety of crocin tablets (20 mg per day for 1 month) on biomedical, hormonal, hematological, and urinary parameters in pre- and post-treatment periods. In their study, crocin tablets did not show major side effects or any change in the above- mentioned factors except decreased mixed white blood cells, amylases, and PTT during trial(39). 

## Limitations

Lack of a placebo group, small sample size, and short follow-up period were the limitations of this study. Further research, taking into account the above- mentioned limitations in this realm, is highly recommended.

## Conclusion

The results of the present study revealed that saffron is effective in the treatment of mild to moderate obsessive compulsive disorder. In addition, this study found that saffron does not have any serious adverse effects in 30 mg/day doses and that saffron is both tolerable and effective in the treatment of mild to moderate depression.
